# Removal of Bromine from Polymer Blends with a Composition Simulating That Found in Waste Electric and Electronic Equipment through a Facile and Environmentally Friendly Method

**DOI:** 10.3390/polym15030709

**Published:** 2023-01-31

**Authors:** Maria Anna Charitopoulou, Lambrini Papadopoulou, Dimitris S. Achilias

**Affiliations:** 1 Laboratory of Polymer and Colours Chemistry and Technology, Department of Chemistry, Aristotle University of Thessaloniki, 54124 Thessaloniki, Greece; 2 Department of Mineralogy-Petrology-Economic Geology, Aristotle University of Thessaloniki, 54124 Thessaloniki, Greece

**Keywords:** WEEE, pyrolysis, soxhlet extraction, brominated flame retardants, TBBPA, XRF

## Abstract

The increasing volume of plastics from waste electric and electronic equipment (WEEE) nowadays is of major concern since the various toxic compounds that are formed during their handling enhance the difficulties in recycling them. To overcome these problems, this work examines solvent extraction as a pretreatment method, prior to thermochemical recycling by pyrolysis. The aim is to remove bromine from some polymeric blends, with a composition that simulates WEEE, in the presence of tetrabromobisphenol A (TBBPA). Various solvents—isopropanol, ethanol and butanol—as well as several extraction times, were investigated in order to find the optimal choice. Before and after the pretreatment, blends were analysed by X-ray fluorescence (XRF) to estimate the total bromine content. Blends were pyrolyzed before and after the soxhlet extraction in order to evaluate the derived products. FTIR measurements of the polymeric blends before and after the soxhlet extraction showed that their structure was maintained. From the results obtained, it was indicated that the reduction of bromine was achieved in all cases tested and it was ~34% for blend I and ~46% and 42% for blend II when applying a 6 h soxhlet with isopropanol and ethanol, respectively. When using butanol bromine was completely eliminated, since the reduction reached almost 100%. The latter finding is of great importance, since the complete removal of bromine enables the recycling of pure plastics. Therefore, the main contribution of this work to the advancement of knowledge lies in the use of a solvent (i.e., butanol) which is environmentally friendly and with a high dissolving capacity in brominated compounds, which can be used in a pretreatment stage of plastic wastes before it is recycled by pyrolysis.

## 1. Introduction

The amount of end-of-life electronic devices has increased significantly over the last few years, due to people’s increased demand for such devices, along with their short lifespan [1]. Plastics originating in waste electric and electronic equipment (WEEE) account for a large percentage, almost 30%, of WEEE and the most abundant are acrylonitrile-butadiene-styrene (ABS), high-impact polystyrene (HIPS), polycarbonate (PC), blends of PC/ABS, polypropylene (PP), etc. [2]. The plastic part of WEEE usually contains various toxic additives, such as brominated flame retardants (BFR) (for instance, polybrominated biphenyls (PBB), tetrabromobisphenol A (TBBPA), etc.), colourants, plasticizers and others [3,4]. BFRs are incorporated into plastics in order to protect people from accidental fires [5], since they reduce plastics’ flammability; among them, TBBPA is the most commonly used in plastics originating in WEEE. However, humans’ exposure to BFR, through inhalation, ingestion or even dermal contact has a negative impact on their health. As a result, taking into account that BFRs are toxic substances, the handling of brominated plastics demands particular attention in order to prevent environmental pollution [6,7]. Chaine et al. [8] recently reviewed the environmental and human health challenges associated with BFRs during the recycling of plastics from WEEE.

Until now, vast amounts of WEEE end up in landfill, causing serious environmental issues by polluting the ground and the groundwater as contaminants leach [9]. In an attempt to eliminate the use of landfill to dispose of plastics, research has focused on recycling methods, which include primary recycling, energy recovery, mechanical recycling and chemical recycling [2,10]. Among them, chemical recycling, and especially pyrolysis, seems to be a promising technique, since it leads to the formation of monomers or secondary valuable materials [2,9,10]. During pyrolysis, plastic waste is converted into liquid, gas and solid residues [11,12,13]. However, when brominated flame retarded plastics are pyrolyzed, the products obtained contain various undesirable brominated compounds that inhibit the reuse of the useful pyrolysis products [14,15]. 

To date, many pretreatment methods, before or during pyrolysis, for the debromination of plastics from WEEE have been investigated [16]. Ma et al. [17] applied single and two-step pyrolysis of waste computer casing plastic with the aim of optimizing the pyrolysis products produced. They concluded that during two-step pyrolysis the bromine content was mainly found in the liquid fraction of the first step, resulting in a high-quality liquid fraction with low bromine content after the second step. Bhaskar et al. [18] made the same observations during a previous study in which they found that the addition of a second pyrolysis step greatly improved the derived liquid fraction, since most of the bromine was transferred into the liquid products of the first step. 

Apart from controlling pyrolysis steps in order to minimize the formation of brominated compounds, many researchers have explored the use of various catalysts. For instance, Wang et al. [19] applied catalytic pyrolysis of waste printed circuit boards using activated alumina and found that in its presence the formation of brominated compounds was reduced. In another work, red mud, limestone and natural zeolite were examined for their efficiency in removing bromine and antimony from pyrolysis oils and it was observed that all of them could lead to bromine reduction, although the red mud produced the best debromination results [20]. In our previous study [21], five different catalysts were investigated for their efficiency in reducing the brominated products formed during pyrolysis of some brominated flame-retarded polymeric blends. According to the results, Fe/Al_2_O_3_, Fe/MgO and MgO led to significant bromine reduction, greater than 55%. 

Other studies have investigated the removal of BFR before pyrolysis. Zhang and Zhang [22] applied solvothermal treatment on plastics that were brominated flame retarded with TBBPA, to obtain bromine-free plastics. For this reason, they used some alcohols—methanol, ethanol and isopropanol—as solvents, due to their non-toxicity. It was observed that TBBPA was eventually removed from the plastics into the solvents, while the structure of the plastics remained the same, meaning they could be recycled. In a recent work [23], solvent extraction before pyrolysis was implemented, with the aim of removing TBBPA from real WEEE, using isopropanol and toluene as solvents. The results indicated that TBBPA could be extracted during this pretreatment. They underlined that despite the fact that the degree of removal was relatively low for the experimental conditions they applied, by adjusting other operation parameters, such as time, the extraction efficiency can be improved. Supercritical carbon dioxide has also been proposed for the extraction of BFRs from WEEE-derived ABS [24]. 

Selection of the optimum solvent is a challenging process since besides its solubility, according to the principles of green chemistry, it should also combine several additional requirements. These include a low impact on the environment; low or no risk to health and safety; low energy requirements for its production, transport, storage, use and disposal; and to be preferably sourced from renewable feedstock [25,26,27]. Of course, finding the optimum solvent that fulfils all these criteria is the ideal, thus many guides have been prepared that rank solvents from least to most hazardous [25]. For instance, the solvent selection guides released by the CHEM21 European consortium [25] provide the user with several suggestions for green solvents that can be used in place of more hazardous ones [25]. Based on these guides, it can be seen that solvents used in the past often go against the principles of green chemistry, particularly in regard to polymer dissolution. Polar solvents such as the alcohols ethanol, isopropanol and n-butanol are listed as environmentally safe in the CHEM21 list and are at the top of the list of green chemicals [28]. Therefore, they were used in this investigation. According to the limited literature data, isopropanol and ethanol have been investigated in some solvothermal pretreatment methods [22,23]. The main difference in this work is the use of varying experimental conditions, such as temperature and extraction time. On the other hand, no data were found in the literature regarding the use of butanol in extracting BFR from WEEE, so its use is also of adequate novelty.

So far, many studies have investigated various pretreatment methods, either before or during pyrolysis, for the debromination of plastics from WEEE; nevertheless, there are still limitations and difficulties that prevent them from being applied industrially. With the aim of overcoming these problems, this study investigates the reduction of bromine focusing on some polymeric blends that consist of ABS, HIPS, PC and PP with a composition that simulates real WEEE, by applying soxhlet extraction as a pretreatment method before pyrolysis. The aim of this study is to remove bromine from the blends before they are subjected to pyrolysis, enabling the recovery of the pure polymers while hindering the formation of the toxic brominated products. Blends are brominated flame retarded using TBBPA, as it is the most common BFR used in WEEE. The main goal of this research is to evaluate the debromination efficiency of different solvents, especially butanol whose use has never been reported in the literature and different extraction times in order to find the optimal combination that can result in the greatest bromine reduction. For the estimation of the total bromine content before and after the pretreatment, X-ray fluorescence (XRF) was carried out. Furthermore, thermal pyrolysis of the blends was conducted using a pyrolyzer combined with a gas chromatographer/mass spectrometer (GC/MS) before and after soxhlet extraction in order to evaluate its effect on the distribution of the products. Fourier transform infrared spectroscopy (FTIR) was applied both on the pure solvents and on the solvents after soxhlet extraction with the aim of finding any additional peaks that could be attributed to the possible capturing of Br (transferring of TBBPA) into the solvents after the pretreatment. Last but not least, FTIR was also performed on the blends before and after the pretreatment in order to observe whether the plastics’ structure was changed at all.

## 2. Materials and Methods

### 2.1. Materials

The polymers used for the preparation of the blends include the most abundant polymers found in WEEE: ABS [(C_15_H_17_N)n, FW = 211.3, batch# 01519EB, melt index: 6 g/10 min], HIPS ((C_8_H_8_)x∙(C_4_H_6_)z, CAS 9003-55-8, lot# 02122CEV, melt index: 6 g/10 min, butadiene content 4%), PC (C_15_H_16_O_2_, CAS 25037-45-0, lot# 07624KHV, melt index: 7 g/10 min) and PP ((CH_2_CH(CH_3_))n, CAS 9003-07-0, batch# 04227KC), all supplied by Sigma-Aldrich (St. Luis, USA). Polymeric blends were prepared in the presence of TBBPA (3, 3′, 5, 5′-tetrabromobisphenol A, CAS 79-94-7), which was the examined BFR and was also purchased from Sigma-Aldrich (St. Luis, MO, USA). 

Several solvents were initially screened in order to select the optimal choice based on their solvency at TBBPA, environmental friendliness, low toxicity [22] and recommendation according to green chemistry principles. The solvents examined were ethanol, propanol, isopropanol, butanol, methanol, THF, toluene, xylene, acetonitrile, cyclohexane, DMF and chloroform. The solvents that were finally selected for the soxhlet extraction experiments were: isopropanol (CAS# 67-63-0, d = 0.78 g/mL, batch# 21.0810404.4800), ethanol (CAS#64-17-5, d = 0.79 g/mL) and butanol (CAS# 71-36-3, d = 0.811 g/mL, batch# 20H114011). The particular selection of the solvents was also based on their solubility parameters. Generally, the similarity in solubility parameters for some solvents and substances (e.g., polymers or BFR) results in these substances being dissolved when using these solvents [29,30]. Therefore, their selection was also based on the fact that they present high solubility for TBBPA and not for the polymers. 

### 2.2. Blends Preparation 

Two polymer blends were prepared using the melt-mixing method in an extruder. After weighing the appropriate amounts of the polymers and TBBPA, they were placed into a twin-screw extruder (Thermo Scientific HAAKE MiniLab, Waltham, MA, USA) at 210 °C and 30 rpm. The first blend (blend I) consisted of 46% ABS, 39% HIPS, 15% PC and 9% TBBPA and the second one (blend II) consisted of 41% ABS, 34% HIPS, 14% PC, 11% PP and 9% TBBPA. The percentages of the polymers were based on the percentages in which they are found in real WEEE [31]. Afterwards, the extrudates that were received were processed into thin films by hot pressing at approximately 200 °C. 

### 2.3. Soxhlet Extraction Procedure 

Solvent-based extraction was carried out using a soxhlet extractor. Each time ~1.3 g of a blend was placed into the thimble in the soxhlet apparatus and the spherical flask was filled with 130 mL of each solvent being examined. The extraction temperature was based on the boiling point of the solvent used. As mentioned above, three different extraction times of 3, 6 and 12 h were applied and compared for their efficiency, in order to find the optimal conditions. Furthermore, three different solvents were examined for their efficiency in removing TBBPA from the polymeric blends.

### 2.4. Analytical Methods

In order to determine whether the structure of the polymeric blends was preserved, FTIR was used both before and after the soxhlet extraction. It was also employed on the pure solvents that were used for the soxhlet extraction and the solvents received to look for any potential new peaks that might have formed as a result of the incorporation of TBBPA into the solvents. A Perkin Elmer FTIR spectrometer, Spectrum One (Shelton, CT, USA), was used for FTIR analysis. Spectra between 4000 and 600 cm^−1^ were received. The system had a 4 cm^−1^ resolution, and each spectrum had 16 scans applied.

To estimate the total bromine content of the blends both before and after the treatments, X-ray fluorescence (XRF) was used. A S4-Pioneer wavelength dispersive spectrometer from the Aristotle University of Thessaloniki’s scanning electron microscopy lab was employed. Before and after the pretreatment, samples of each blend were cut into small pieces that were suitable for the measurements. 

For the purpose of identifying the pyrolysis products, the blends were thermally pyrolyzed using a pyrolyzer coupled with a gas chromatograph/mass spectrometer (GC/MS). In order to assess the impact of the soxhlet extraction on the distribution of the products, pyrolysis was conducted at 400 °C both before and after the extraction. Helium was used as the purge gas in pyrolysis tests, and the sample weight was 1 mg each time. Details on the pyr-GC/MS program can be found in Charitopoulou et al. [32]. Shimadzu post-run software was used to interpret the received chromatograms (NIST 17 Library). Prior to pyrolysis, the mass spectrometer’s settings included both the SCAN mode, which scanned the entire spectrum (both before and after the soxhlet extraction) for unknown substances, and the selected ion monitoring (SIM) mode. SIM targeted to specific ions (250, 252 and 254) for the determination of dibromophenol peak (before and after the soxhlet extraction), since it was found that this compound was formed during the thermal pyrolysis of the blends.

## 3. Results and Discussion

### 3.1. Effect of Solvent Extraction on the Polymer Structure in the Blends

**Figure 1:**
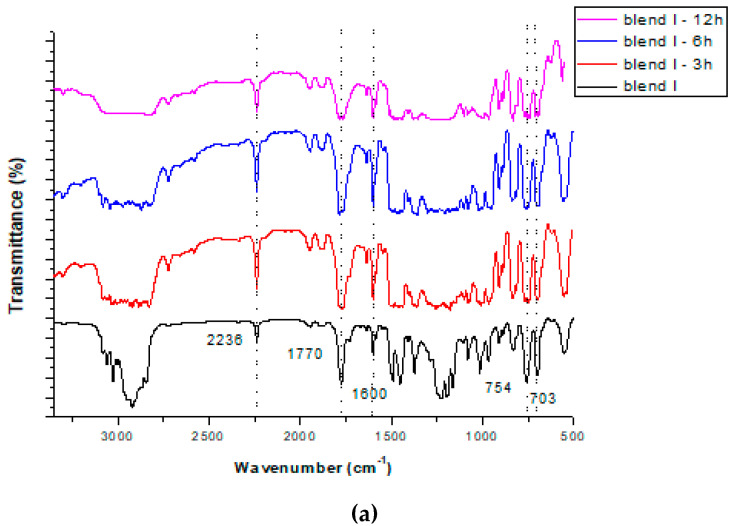

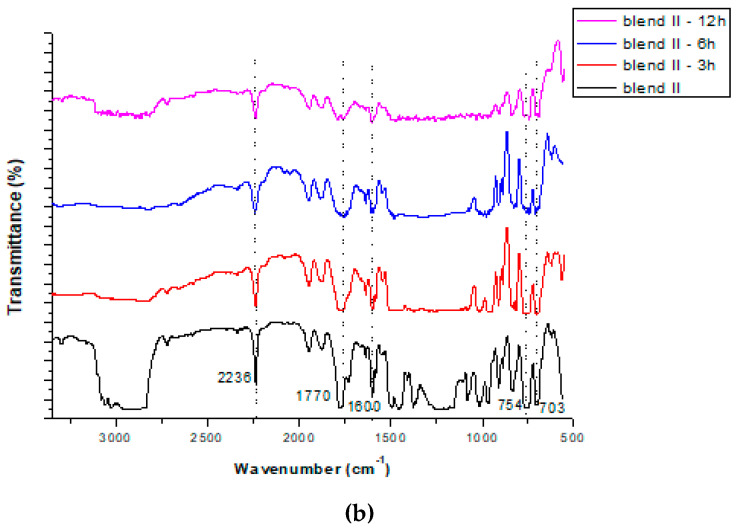
FTIR spectra of the two polymeric blends: blend I (**a**) and blend II (**b**) before pretreatment and after soxhlet extraction with isopropanol at different extraction times: 3, 6 and 12 h.

**Figure 2:**
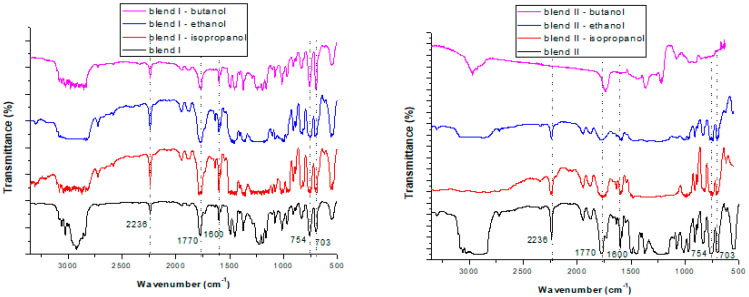
FTIR spectra of the two polymeric blends: blend I (**left**) and blend II (**right**) before pretreatment and after soxhlet extraction for 6 h using different extraction solvents: isopropanol, ethanol and butanol.

Initially, it was investigated whether the solvent extraction method affected the structure of the polymers in the blends. As can be seen in Figure 1 and Figure 2, similar FTIR spectra were obtained before and after soxhlet extraction in all conditions (with differing extraction times and solvents used) that were tested. Specifically, in almost all cases a band at 2236 cm^−1^ was observed due to the acrylonitrile units in ABS (v_C-N_). The band at 1770 cm^−1^ was attributed to the presence of PC (v_C = O_). A band at 1600 cm^−1^ was obtained in all cases due to aromatic ring stretching vibrations (v_C = C_). In almost all cases bands at 754 cm^−1^ and 703 cm^−1^ were obtained, due to due to C-H bending, attributed to the styrene units in ABS and HIPS, respectively. Consequently, the extraction, regardless of the solvent used or the extraction time applied, did not affect the structure of the polymers in the blends. This finding concurs with other findings in the literature. For instance, Zhang and Zhang [22] found that polymers’ structures were unchanged after being subjected to solvothermal treatments, as shown by the application of FTIR analysis. Likewise, in our previous study [33], FTIR analysis was conducted on polymeric blends before and after microwave-assisted extraction and it was shown that the extraction did not alter their structure, which was maintained. This is a very important observation, since when applying a pretreatment—debromination method to brominated plastics from WEEE—it is desirable to remove the BFR incorporated into the polymers without influencing their structure.

**Figure 3:**
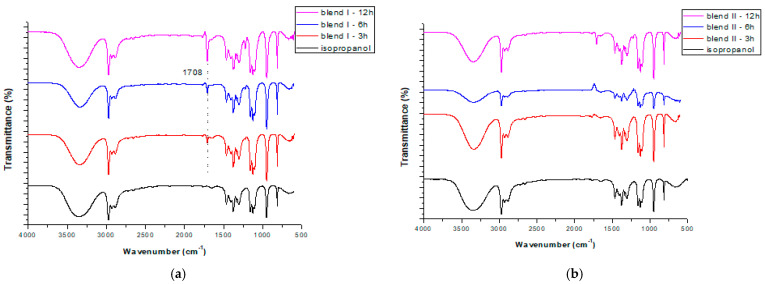
FTIR spectra of the pure isopropanol and isopropanol received after different extraction times, at 3, 6 and 12 h soxhlet extraction: in the case of blend I (**a**) and blend II (**b**).

**Figure 4:**
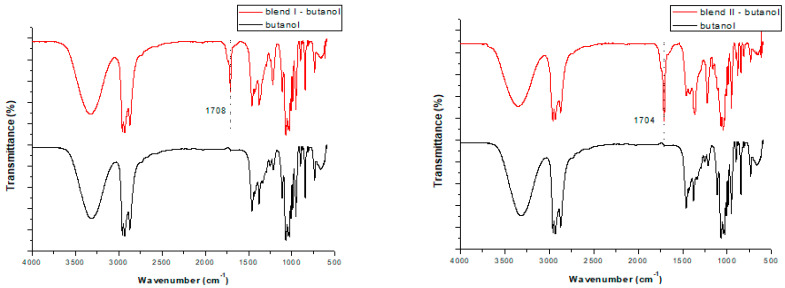
FTIR spectra of the pure butanol and butanol received after applying 6 h soxhlet extraction in the cases of blend I (**left**) and blend II (**right**).

When using isopropanol as the extractive solvent and focusing on the efficiency of the different extraction times (3, 6 and 12h), for both blends studied two extra peaks were formed: one strong peak (Figure 3) at ~1710 or ~1704 cm^−1^ in the cases of blend I and blend II, respectively; and one peak of very low intensity at ~605 cm^−1^ for both blends. The latter peak cannot be seen in Figure 3, since additional enlargement would be required. These two additional peaks are attributed to the transference of TBBPA from the blends to the solvents during soxhlet extraction, as they were not formed initially (without the pretreatment). The same observations were made when focusing on the efficiency of the different solvents when applying a 6 h extraction and using butanol as the extractive solvent (Figure 4). As a result, these two peaks can be considered an indication that soxhlet extraction was efficient in removing bromine (TBBPA) from the polymeric blends, regardless of the type of the solvent used or the time applied for the extraction.

**Figure 5:**
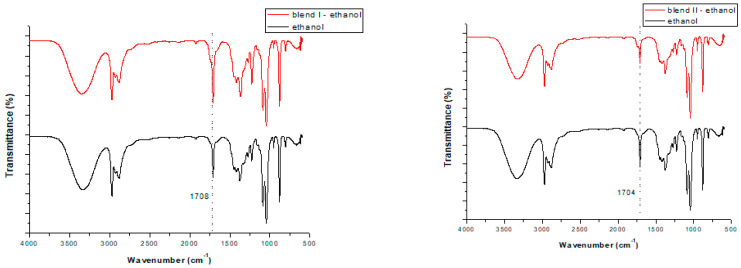
FTIR spectra of the pure ethanol and ethanol received after applying 6 h soxhlet extraction for blend I **(left**) and blend II (**right**).

Nevertheless, when using ethanol as the extractive solvent (Figure 5), the peaks at ~1708 (or ~1704 cm^−1^) and ~605 cm^−1^ cannot be attributed with certainty to the transference of TBBPA from the blends to the solvents, due to the fact that these peaks pre-existed in the case of the pure solvent. Consequently, in the case of ethanol, since these two peaks are not considered an indication that bromine has been removed from the polymeric blends, data from the other methods are required in order to prove its efficiency as an extractive solvent.

### 3.2. Bromine Content before and after Soxhlet Extraction—XRF Results

For the estimation of the total bromine content in the blends before and after the pretreatment, XRF analysis was undertaken. For each blend, many random samples were analysed to assure reliable conclusions and the mean values were calculated. It should be underlined here that all initial values of percentage weight for both blends were very close, which is indicative that they were suitably homogeneous, since the bromine content was almost the same in all samples for each blend. 

In the case of blend I, the initial measurement was 5.528 wt.% bromine and for blend II it was 6.026 wt.%. Table 1 presents the results after applying soxhlet extraction, using isopropanol as the solvent and studying the efficiency of the different extraction times. It was observed that for both blends and all extraction times tested the bromine content was reduced, even when applying soxhlet extraction for just 3 h. Nevertheless, the extraction time strongly affected the bromine content, with the reduction of bromine increasing over time. 

As regards the efficiency of the different solvents used for the extraction, apart from isopropanol, ethanol and butanol were also investigated by applying 6 h soxhlet extraction process. From the results in Table 2, it can be seen that butanol was the best solvent used, leading to the complete removal of all bromine in the case of blend I and to an almost complete removal (98% reduction) in the case of blend II. The differences between isopropanol and ethanol were almost negligible. Similar results for isopropanol and ethanol were observed during another pretreatment method (solvothermal) by Zhang and Zhang [22], who also observed insignificant differences in these solvents’ efficiency at removing TBBPA. The greater debromination efficiency of butanol may be attributed to its lower solubility in comparison with isopropanol and ethanol. Moreover, it is known that the extent of the similarity in a given situation determines the extent of the interaction [34]. The same cannot be said of the total or Hildebrand solubility parameter [34]. Ethanol and nitromethane, for example, have similar total solubility parameters (26.1 vs. 25.1 MPa^1/2^, respectively), but their affinities are quite different. Ethanol is water soluble, whereas nitromethane is not [34]. In our case, isopropanol and ethanol are miscible in water, whereas butanol presents limited miscibility in water (i.e., 0.11). TBBPA has a low water solubility (i.e., 0.72). Thus, it seems that TBBPA has a similar affinity with butanol.

### 3.3. Pyrolysis Results

**Figure 6:**
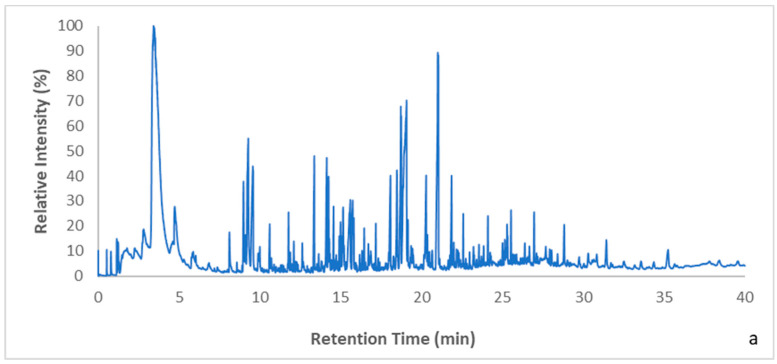

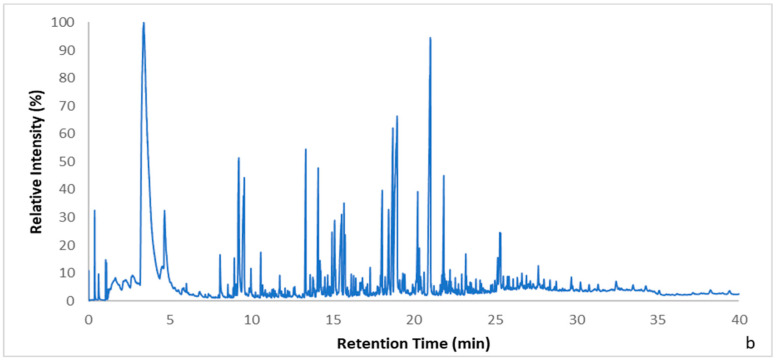
GC/MS chromatograms after pyrolysis of the blends without pretreatment for blend I (**a**) and blend II (**b**).

The products obtained from the pyrolysis of the blends, both before or after the pretreatment, were recorded in a Py-GC/MS device. As can be seen from Figure 6a,b the GC/MS chromatograms of the products derived from the pyrolysis of the two polymeric blends without pretreatment are quite similar. During pyrolysis of both blends, various aromatic hydrocarbons were produced with one, two or three aromatic rings, such as styrene (monomer), 1,3-diphenylpropane (styrene dimer) and 1,3,5-triphenyl-cyclohexane (styrene trimer). Nitrogenated compounds including benzenebutanenitrile, 3-phenyl-2-pentenenitrile, etc., and phenolic compounds such as phenol, p-isopropylphenol, p-isopropenylphenol, 4-(1-methyl-1-phenylethyl)phenol and 3,4′-isopropylidenediphenol were also formed. All of these are valuable secondary products that can be used as feedstock or for the production of other useful compounds. Their formation is attributed to the degradation of the polymers present in the blends; however, the formation of phenols is not only due to the degradation of PC, but also due to the presence of TBBPA, which enhances the formation of such compounds [35]. Apart from these useful products, some brominated compounds, dibromophenol and bromomethyl benzene, were also obtained due to the degradation of TBBPA. The intensity of these peaks was very low, since TBBPA is present in small quantities in the blends, contrary to the polymers, which are abundant in both blends.

As mentioned previously, apart from applying the scan mode in the mass spectrometer’s settings, a SIM analysis was also used in order to ensure the presence of the mentioned brominated compounds. When targeting the ions (250, 252 and 254) used to identify dibromophenol, its presence was confirmed. However, when targeting specific ions used to identify bromomethyl benzene, its presence could not be confirmed. As a result, in this study SIM analysis was only focused on the dibromophenol peak before and after soxhlet extraction in order to evaluate the process’s efficiency on the total peak area. 

**Figure 7:**
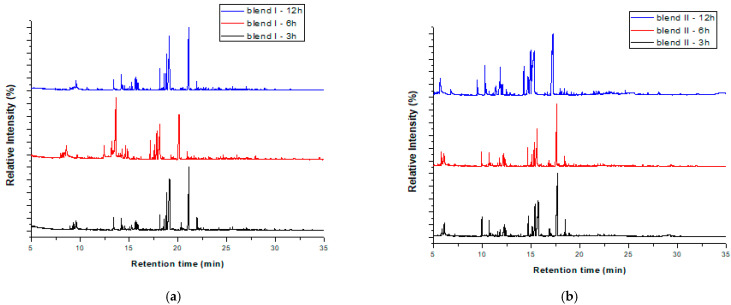
GC/MS chromatograms after pyrolysis of the blends after soxhlet extraction with isopropanol for 3, 6 and 12 h for blend I (**a**) and blend II (**b**).

**Figure 8:**
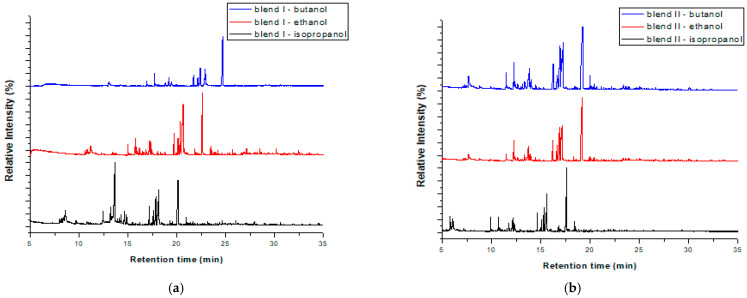
GC/MS chromatograms after pyrolysis of the blends after soxhlet extraction for 6 hr. with isopropanol, ethanol and butanol for blend I (**a**) and blend II (**b**).

The blends isolated after applying soxhlet extraction with isopropanol for 3, 6 and 12 h and isopropanol, ethanol, butanol for 6 h were also subjected to pyrolysis in the same lab-scale pyrolyzer. The products obtained were detected with GC-MS and the results are illustrated in Figure 7a,b and Figure 8a,b, respectively. The chromatograms of each blend were almost the same, regardless of the extraction time and the extractive solvent; the main differences were the retention time the peaks were obtained and the intensity of some peaks. In addition, it was observed that most of the products that were formed before the pretreatment method continued to be formed after it. Specifically, hydrocarbons such as styrene (monomer), 1,3-diphenylpropane (styrene dimer) and 1,3,5-triphenyl-cyclohexane (styrene trimer) continued to be produced at the same retention times as those recorded without the pretreatment. The same observation was made for various other compounds, including nitrogenated compounds (e.g., benzenebutanenitrile) and phenolic compounds, such as 4-(1-methyl-1-phenylethyl)phenol and 3,4′-isopropylidenediphenol. The fact that the products’ distribution was not affected before and after soxhlet extraction indicates that the polymers’ structure is maintained during this pretreatment. This is in accordance with the FTIR results.

It should be mentioned that during pyrolysis of the blends after the soxhlet extractions no brominated compounds could be identified when applying the SCAN mode, in contrast with the results obtained from the initial pyrolysis of the blends, where brominated compounds were obtained. This can be attributed to the low content of bromine left in the blends after the pretreatment and indicates that soxhlet extraction is an efficient debromination method. Nevertheless, since this is only a qualitative observation, XRF and SIM analysis were conducted in order to evaluate the bromine reduction more accurately.

**Figure 9:**
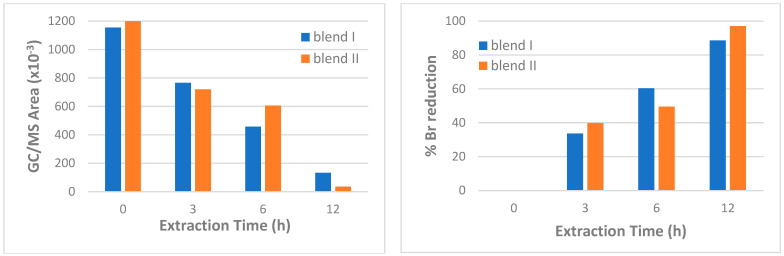
Effect of extraction time on GC/MS area of dibromophenol peak (**left**) and on percentage bromine reduction (**right**), for blend I and blend II.

During SIM analysis and focusing on the efficiency of the extraction time when using isopropanol as solvent, it was observed that for both blends, the area of dibromophenol peak was decreased for all times tested (Figure 9). The reduction increased as time increased from 3 to 12 h. Specifically, in the case of blend I, the reduction was ~34% in the case of a 3 h soxhlet with isopropanol, becoming ~60% in the case of 6 h soxhlet extraction and, finally, ~89% for the 12 h soxhlet extraction. Likewise, for blend II the reduction after 3 h soxhlet extraction was ~39%, becoming ~50% after 6 h and, finally, increasing to ~95% after 12 h extraction. The same trend was observed in the case of the different extractive solvents (isopropanol, ethanol and butanol), when applying 6 h soxhlet extraction. Specifically, for both blends, bromine reduction was observed for all solvents examined; butanol was the best solvent, leading to a complete removal of bromine (100% reduction). The results are in accordance with the XRF results. Nevertheless, when using SIM method the reduction seemed much bigger than that observed from XRF analysis. This was expected and is due to the fact that when applying the former method attention is paid only to the dibromophenol peak, excluding any other bromo-compounds that might be formed and which may be below the detection limit of the method. On the other hand, the results from XRF are based on the total bromine content estimated for each sample, and so are considered as more reliable.

## 4. Discussion

As mentioned previously, the increasing volume of end-of-life plastics from WEEE, along with the possible formation of various toxic brominated products during their recycling, necessitates the application of a pretreatment method prior to recycling. The present study therefore investigated soxhlet extraction before pyrolysis with the aim of removing bromine from some polymeric blends that simulate WEEE. The examined BFR was TBBPA. 

Three different solvents—isopropanol, ethanol and butanol—as well as different extraction times were examined to find the optimal combination of solvent and time. The results obtained both from XRF and SIM analyses showed that all extraction times applied led to bromine reduction, and it was observed that when the time was doubled the debromination efficiency also doubled. For instance, in the case of blend I, the debromination was ~17% according to XRF analysis after 3 h soxhlet extraction with isopropanol, rising to 34% after a 6 h process with the same solvent. 

In addition, it was indicated that all solvents used resulted in an efficient reduction of bromine, ranging from ~34% to 46% for both blends when applying 6h soxhlet with isopropanol and ethanol. However, the complete elimination of bromine was achieved after 6 h soxhlet extraction with butanol, enabling the subsequent recovery of monomers and other valuable products formed during the pyrolysis of the pure plastics. 

In all cases tested, the XRF results were in agreement with the SIM analysis results. Nevertheless, the latter method led to higher results for bromine reduction for all cases, which can be attributed to the fact that SIM analysis focused only on dibromophenol area, whereas XRF took into account the entire mass of the sample and thus estimated the total bromine content of each sample. 

The most important finding was that when using butanol, which is a novel choice of solvent, brominated plastic samples can become bromine-free plastics without changing their polymeric structures, as proved by FTIR analysis. This means they can be recycled easily. 

## 5. Conclusions

This work investigated soxhlet extraction as a pretreatment method before pyrolysis, for the removal of brominated compounds such as TBBPA from polymeric blends with a composition that simulates WEEE. Various extraction times (3, 6 and 12 h) and extractive solvents (isopropanol, ethanol and butanol) were examined in order to optimize the method. Various techniques, including XRF (for estimating the total bromine content), pyrolysis (as a method for recycling method polymers from WEEE), FTIR (for the characterization of the chemical structure of the solvents and the polymeric blends) were applied in order to evaluate the efficiency of extraction.

It was found that bromine levels were reduced in all cases tested (times and solvents), and that in the case of a 6 h extraction process with butanol, bromine was completely eliminated, being reduced by almost 100%. Apart from butanol, which was the most efficient solvent used, isopropanol and ethanol also led to a significant reduction in bromine, with both blends tested resulting in a ~30–40% bromine reduction. As for the efficiency of the different extraction times, it was observed that even when applying a 3 h soxhlet extraction, bromine reduction took place. Generally, as time increased the debromination efficiency also increased. These findings were verified by both XRF and SIM analysis. Last but not least, FTIR analysis showed that the structure of the polymers in the blends was maintained during soxhlet extraction, with the spectra observed before and after the extraction similar under all conditions investigated. 

As a result, we can conclude solvent extraction is a fruitful method for the removal of BFR from plastics, and that butanol may lead to the complete removal of TBBPA from the polymers without changing their structure. The findings of this study, which is the first step of an ongoing project on the recycling of plastics from real WEEE, may provide insights for future studies, for example, using butanol as an extractive solvent for the debromination of brominated flame-retarded plastics collected from real WEEE, enabling the subsequent recycling of the pure polymers. Another future project currently under investigation focuses on applying the same method on a larger scale, where greater amounts of waste are treated with lower quantities of solvents (butanol). 

## Figures and Tables

**Table 1:** Bromine content after using isopropanol as solvent and applying different extraction times.

Extraction Time (h)	Blend Name	wt.% Br Measured	% Br Reduction
3	Blend I	4.613	17
	Blend II	4.493	25
6	Blend I	3.67	34
	Blend II	3.243	46
12	Blend I	1.23	78
	Blend II	0.661	89

**Table 2:** Bromine content after applying a 6 h soxhlet extraction using different extractive solvents.

Extractive Solvent	Blend Name	wt.% Br Measured	% Br Reduction
Isopropanol	Blend I	3.67	34
	Blend II	3.243	46
Ethanol	Blend I	3.646	34
	Blend II	3.494	42
Butanol	Blend I	BDL *	100
	Blend II	0.0986	98

* BDL: below detection limit.

## Data Availability

Data are available on request.
